# HOSPITAL-IN-HOME: IMPLEMENTATIONS IN THE VA HEALTH CARE

**DOI:** 10.1093/geroni/igad104.0535

**Published:** 2023-12-21

**Authors:** Jennifer Sullivan, Emily Franzosa, Dayna Cooper

## Abstract

The Department of Veterans Affairs (VA) Hospital-In-Home (HIH) program delivers patient-centered, acute-level hospital care at home within a single-payer integrated health-care system. Compared to inpatient care, HIH has demonstrated improved patient safety, effectiveness, and patient and caregiver satisfaction. As of 2022, there were eleven HIH sites across the VA, evidence of modest adoption. This symposium presents findings from the first year of a 4-year project that aims to conduct an in-depth inquiry in to the most effective ways to implement, adapt and sustain HIH across the VA. We will present an overview of the VA HIH program, describe one HIH program in-depth, present a birds-eye-view of the national program, present comparisons of sites’ implementations in terms of structure, patient characteristics and outcomes, and conclude with an implementation-science framework to examine program adoption, adaptations, successes and challenges. National leadership will provide perspectives on management, planning and policy of Hospital-In-Home.

